# Gaps in data collection for sex and gender must be addressed in point prevalence surveys on antibiotic use

**DOI:** 10.3389/frabi.2023.1154506

**Published:** 2023-03-17

**Authors:** Lynn Lieberman Lawry, Niranjan Konduri, Nkatha Gitonga, Reuben Kiggundu, Mame Mbaye, Andy Stergachis

**Affiliations:** ^1^ Overseas Strategic Consulting, Philadelphia, PA, United States; ^2^ United States Agency of International Development (USAID) Medicines, Technologies and Pharmaceutical Services Program (MTaPS), Management Sciences for Health, Arlington, VA, United States; ^3^ United States Agency of International Development (USAID) Medicines, Technologies and Pharmaceutical Services Program (MTaPS), Management Sciences for Health, Nairobi, Kenya; ^4^ United States Agency of International Development (USAID) Medicines, Technologies and Pharmaceutical Services Program (MTaPS), Management Sciences for Health, Kampala, Uganda; ^5^ United States Agency of International Development (USAID) Medicines, Technologies and Pharmaceutical Services Program (MTaPS), Management Sciences for Health, Dakar, Senegal; ^6^ Departments of Pharmacy and Global Health, School of Pharmacy and School of Public Health, University of Washington, Seattle, WA, United States

**Keywords:** sex-disaggregation, gender, point prevalence survey (PPS), antimicrobial resistance (AMR), antimicrobial use (AMU), health equity

## 1 Introduction

### 1.1 Antimicrobial resistance and antimicrobial stewardship

Antimicrobial resistance (AMR) is a leading global public health threat (Murry et al., 2022). In 2019, there were 1.27 million deaths attributable to bacterial AMR (Murry et al., 2022). Antimicrobial resistance leads to longer illnesses, increased treatment costs, and increased mortality (World Bank, 2017). AMR presents a threat to the global economy estimated to cost more than $1 trillion annually by 2050 (World Bank, 2017). Antimicrobial resistance is accelerated by the misuse and overuse of antimicrobials, as well as poor infection prevention and control (IPC). But there are other AMR risks including the impact of sex, gender, and systemic inequities (World Health Organization, 2018). Antimicrobial stewardship (AMS) helps antimicrobials remain effective by decreasing their inappropriate use, a contributor to increasing AMR to common first-line antimicrobials (World Health Organization, 2018; Brandl et al., 2021; Murry et al., 2022; World Health Organization, 2022). Overprescribing, inappropriate use, over dispensing of antimicrobials by health workers, nonadherence with treatment courses or self-treatment, poor quality antimicrobials, and poor IPC, hygiene, and sanitation practices in healthcare facilities all contribute to the global AMR crisis (World Health Organization, 2018; Brandl et al., 2021; Murry et al., 2022; World Health Organization, 2022). AMR threatens the effective prevention and treatment of infections and undermines health gains globally as antimicrobials become less effective (World Health Organization, 2018). There is a dearth of information on antimicrobial consumption and use especially in low- and middle-income countries (World Health Organization, 2018; World Health Organization, 2022). Standardized monitoring of antimicrobial use (AMU) underpins the effective implementation and success of AMS interventions in combatting AMR (, ; Samverkan mot Antibiotikaresistens (STRAMA), 2006; Seaton et al., 2007; Versporten et al., 2016; European Centre of Disease Control and Prevention (ECDC), 2016-2017; World Health Organization, 2019; Kakkar et al., 2021; European Antimicrobial Resistance Surveillance System (EARSS), 2022). Point prevalence survey tools, are standardized surveillance tools to collect information from medical records of hospitalized patients (, ; Samverkan mot Antibiotikaresistens (STRAMA), 2006; Seaton et al., 2007; Versporten et al., 2016; European Centre of Disease Control and Prevention (ECDC), 2016-2017; World Health Organization, 2019; Kakkar et al., 2021; European Antimicrobial Resistance Surveillance System (EARSS), 2022). The antimicrobial information collected include, but are not limited to, the substance name, dosage, route of administration, indication and category of patients by specialty and healthcare facility (, ; Samverkan mot Antibiotikaresistens (STRAMA), 2006; Seaton et al., 2007; Versporten et al., 2016; European Centre of Disease Control and Prevention (ECDC), 2016-2017; World Health Organization, 2019; Kakkar et al., 2021; European Antimicrobial Resistance Surveillance System (EARSS), 2022). The overall aim of the tools is to support policymakers and practitioners to improve AMU as a part of an AMS program (, ; Samverkan mot Antibiotikaresistens (STRAMA), 2006; Seaton et al., 2007; Versporten et al., 2016; European Centre of Disease Control and Prevention (ECDC), 2016-2017; World Health Organization, 2018; World Health Organization, 2019; Kakkar et al., 2021; European Antimicrobial Resistance Surveillance System (EARSS), 2022; World Health Organization, 2022). These data are used to raise awareness of AMU in hospitals, build the capacity of healthcare staff in monitoring and evaluation and to identify problems of antimicrobial prescribing and use (, ; Samverkan mot Antibiotikaresistens (STRAMA), 2006; Seaton et al., 2007; Versporten et al., 2016; European Centre of Disease Control and Prevention (ECDC), 2016-2017; World Health Organization, 2019; Kakkar et al., 2021; European Antimicrobial Resistance Surveillance System (EARSS), 2022). By doing so, it is then possible to set up priorities to address any gaps in AMS. However, these tools have gaps. First we will summarize sex and gender impacts on AMR.

### 1.2 Sex impacts on AMR

Sex, a biological classification, is determined by both physiological and biological factors that define males, females, and intersex individuals. These factors include chromosomal, hormonal, and anatomical characteristics, that is, external reproductive organs, and internal genitalia (Soldin and Mattison, 2009). On average, males and females differ in body weight, size of vital organs like the liver and kidneys, total body water, extracellular and intracellular water, total volume of blood, plasma, and red blood cells (Soldin and Mattison, 2009). Differences between males and females are also apparent in hormones, kidney function, hepatic function, gastric emptying/intestinal motility, and cardiac output (Soldin and Mattison, 2009). These differences affect medicine absorption, distribution, metabolism, and elimination of medicines and raise the risk for adverse drug events including overdoses and AMR among females when compared to males (Soldin and Mattison, 2009).

### 1.3 Gender impacts on AMR

Gender is defined as the socially constructed roles, behaviors, activities, and attributes that a given society considers appropriate for men, women, transgender, or non-binary individuals (Soldin and Mattison, 2009). Gender identity and expression are not always aligned with sex assigned at birth. These norms can influence prescribing practices of healthcare providers towards men and women. And they shape health needs and medication use through access to and utilization of health services, decision-making power, economic status, education, occupational choice, access to and control over resources and high-risk behaviors in relation to the seeking and use of antimicrobials (Action on Antibiotic Resistance, 2020; Asiimwe et al., 2021).

### 1.4 PPS tools

Of the PPS tools used for assessing AMU, except for one aspect of the WHO PPS, all have gaps for data collection and the analysis of associations between sex, gender, pregnancy and AMU and AMR. (**Table 1**) (, ; Samverkan mot Antibiotikaresistens (STRAMA), 2006; Seaton et al., 2007; Versporten et al., 2016; European Centre of Disease Control and Prevention (ECDC), 2016-2017; World Health Organization, 2019; Kakkar et al., 2021; European Antimicrobial Resistance Surveillance System (EARSS), 2022) The Antimicrobial Rational Assessment Tool (AmRAT) is the only instrument that did not include sex or gender and allow for the ability to disaggregate data by sex or gender (Kakkar et al., 2021). In the WHO PPS tool, the coded values for ‘gender’ are male, female and transgender (World Health Organization, 2019). Male and female are categories for ‘sex’; however, transgender is a category for ‘gender’ therefore these variables are incorrectly categorized in the PPS methodology (World Health Organization, 2019). And it is unclear what the relevance of coding simply ‘transgender’ adds to medical information if the category of transgender is not clarified (i.e., transgender women or transgender man) especially since only transgender men (biologically female) can become pregnant. Pregnancy and the related pharmacokinetic differences are important variables not currently specified in any of the PPS tools especially given elevated rates of antibiotic use among pregnant individuals (Soldin and Mattison, 2009). Even with data collected among adolescents, a time when hormonal therapy is started for gender transition, the Antibiotic Resistance and Prescribing in European Children ARPEC/GARPEC does not collect gender or pregnancy data (Versporten et al., 2016).

**Table 1:** Point Prevalence Survey Tools and Variables for Sex, Gender, and Pregnancy.

PPS Survey Tool	Coded Variables for Sex	Coded Variables for Gender	Coded Variable for Pregnancy
Antimicrobial Rational Assessment Tool (AmRAT) (Kakkar et al., 2021)	No	No	No
Antibiotic Resistance and Prescribing in European Children ARPEC and Global APRPEC (GARPEC) (Versporten et al., 2016)	Yes, male/female	No	No
European Antimicrobial Resistance Surveillance System (EARSS) (2022)	Yes, male/female/other/unknown miscoded as gender	No	No
European Centre of Disease Control and Prevention (ECDC) (2016-2017)	Yes, male/female	No	No
Glasgow Antimicrobial Audit Tool (GAAT) (Seaton et al., 2007)	Yes, male/female miscoded as gender	No	No
Global Point Prevalence Survey (GLOBAL-PPS) (,)	Yes, male/female/unknown	No	No
Samverkan mot Antibiotikaresistens (STRAMA) (Samverkan mot Antibiotikaresistens (STRAMA), 2006)	Yes, male/female	No	No
WHO PPS (World Health Organization, 2019)	Yes, male/female miscoded as gender	Yes, transgender but not transgender man/women	No

## 2 Discussion

### 2.1 Why adding a sex variable is important

The sex variables utilized should be male, female, and intersex. Intersex should be added as a variable under ‘sex’ because of the unique biology of intersex individuals and the potential for different AMR risk given they might have both male and female hormones (Soldin and Mattison, 2009). Estrogen and testosterone levels change with age (Klein, 2000). Therefore age-, hormone- and sex-disaggregated data are important to determine immune status and AMR risk throughout lifecycles as the ability to mount an immune response to bacterial infections over time and with exogenous supplement of hormone therapy such as in post-menopausal persons, gender transition or those with reproductive cancers changes (Klein, 2000; Soldin and Mattison, 2009; World Health Organization, 2018). Females are prescribed more antimicrobials in primary care putting them at increased risk for AMR especially when they are treated for urinary tract infections (UTI) which are becoming highly resistant to first line antibiotics due to antibiotic overuse contributing to increasing severe illness, hospitalizations, and higher mortality (World Health Organization, 2018; Trautner et al., 2022). More than half of females, in their lifetime have been treated for an UTI and half of those treated on multiple occasions (Geerlings, 2016). Given the high exposure to antimicrobials for females, including for frequent treatment of urinary bacteriuria during pregnancy, and the possible horizontal transmission of antimicrobials to infants, a sex variable in PPS tools for those who are biologically female and a pregnancy variable will help us better understand all aspects of AMR among females, pregnant individuals, and their infants to address antibiotic overuse at all levels of healthcare (Soldin and Mattison, 2009; Smaill and Grivell, 2014; World Health Organization, 2018; Action on Antibiotic Resistance, 2020; Brandl et al., 2021; World Health Organization, 2022).

Physiologic or pharmacokinetic changes during pregnancy and lactation affect drug metabolism and elimination. Pregnancy, abortion, and childbirth increases the risk of AMR especially if these events happen in healthcare settings without hygienic conditions (World Health Organization, 2018). Females who undergo caesarean section have up to a 20-fold greater risk for infection and infectious morbidity compared with those who have a vaginal birth (Action on Antibiotic Resistance, 2020). Thus, surgical site infections and prolonged use of post-surgical urinary catheters can lead to antimicrobial overuse and AMR (World Health Organization, 2018; Action on Antibiotic Resistance, 2020). Yet as a sex factor, pregnancy status is rarely collected as a part of sex-disaggregated data or included in AMR-related reporting including PPS data. This has a limiting effect for understanding sex-mediated dynamics of disease, understanding vulnerable groups, understanding horizontal transmission, and of appropriate sex-responsive responses to disease and treatment.

### 2.2 Why adding a gender variable is important

Gender should be added to all tools and coded as woman, man, transgender woman (male at birth) and transgender man (female at birth), non-binary, and unknown. As such, transgender data could be collapsed into ‘sex at birth’ data to create more complete sex-disaggregated data. And by using ‘sex at birth’, this may be the way around barriers posed by governments that criminalize sexual and gender minorities and where data might not be collected on gender (Klein, 2000; Soldin and Mattison, 2009; Smaill and Grivell, 2014; World Health Organization, 2018; Murry et al., 2022; World Health Organization, 2022). Looking at gender from a biological perspective (sex at birth) is important and may help to overcome the social issues and or stigma associated with capturing these data. Adding more descriptive variables or categories to gender will allow for improved interpretation of data, especially of transgender individuals given that hormones (innate and/or exogenous) play a role in AMR (Kakkar et al., 2021). Furthermore, gender helps us understand contextualized gendered norms and behaviors that increase exposures to infection and/or the ability to access and/or overuse of antimicrobials. Gender also tells us about the differences in education, economic status and decision-making power that can impact pre-hospital medication use.

### 2.3 Gender norms and behaviors

Gender-based behaviors in handwashing give men a two-fold increase in AMR infections compared with women (Brandl et al., 2021). In pharmacy settings, there is gender bias in the delivery and/or acceptance of antibiotic stewardship recommendations given by women versus those given by men (Vaughn et al., 2022). Men, who more commonly have high-risk behavior, have a two-fold increase in self-medicating with antimicrobials and not finishing a full course of antimicrobials in comparison to women (Zanichelli et al., 2019; Action on Antibiotic Resistance, 2020). Once hospitalized, being able to gender-disaggregate PPS data by gender, helps us understand AMR risk more clearly and utilize these data to develop strategies to address AMR (Jones et al., 2022). Globally, women comprise 70% of the frontline health care workforce, putting them at higher rates of exposure to infectious diseases and needle-stick injuries all compounded by gender norms that make them the caregivers at home and in the community (World Health Organization, 2018; Action on Antibiotic Resistance, 2020). And where gender norms are present that lead to gender-based violence, this put women and sexual and gender minorities at risk of increased use of antimicrobials and AMR; thus, there are many gender norms and factors pre-hospital that will impact hospital AMU and AMR (World Health Organization, 2018). If women contract an antimicrobial-resistant infection, they may be less likely to receive or less able to afford the needed first- and second-line treatments, especially in healthcare settings where the patient is required to purchase their antimicrobials (World Health Organization, 2022). And where there is gender discrimination, sexual and gender minorities will be less likely to seek treatment for sexually transmitted infections or they may self-treat leading to AMR (World Health Organization, 2018).

## 3 Conclusion

Sex and gender are important factors that impact antimicrobial resistance. These variables should be included in PPS methodologies for antibiotic use studies in hospitals to help us understand the pre-hospital risks. Even the AMR global consultancy report published this year, missed an opportunity to discuss relevant sex and gender impacts on AMR and is not in alignment with other WHO guidance on sex and gender equity for health in general and AMR (World Health Organization, 2018; World Health Organization, 2022). Given WHO’s priority to equitably address AMR, all antibiotic PPS survey instruments should be updated to include these data in the PPS methodology, ensuring that sex and gender are collected and recorded in medical records (, ; Samverkan mot Antibiotikaresistens (STRAMA), 2006; Seaton et al., 2007; Versporten et al., 2016; European Centre of Disease Control and Prevention (ECDC), 2016-2017; World Health Organization, 2018; World Health Organization, 2019; Kakkar et al., 2021; European Antimicrobial Resistance Surveillance System (EARSS), 2022; Jones et al., 2022).

## Author contributions

LL and NK take responsibility for the integrity of the manuscript. Manuscript concept: LL, NK. Drafting of the manuscript: LL, NK, NG, RK, MM, AS. Critical revision of the manuscript for important intellectual content: LL, NK, NG, RK, MM, and AS. Administrative, technical, or material support: LL and NK. Manuscript supervision: LL. All authors contributed to the article and approved the submitted version.
